# Supplementary material for: The interactome of intact mitochondria by cross-linking mass spectrometry provides evidence for coexisting respiratory supercomplexes
Source: Mol Cell Proteomics. 2017 Dec 8;17(2):216–32. doi: 10.1074/mcp.RA117.000470 (PMC5795388; doi:10.1074/mcp.RA117.000470)
Supplement: Supplemental Data [file supp_RA117.000470_133922_0_supp_23978_fzffwf.zip › spectra_annotation/mito_spectra_annotation/326-1-7-1-5-1.pdf]

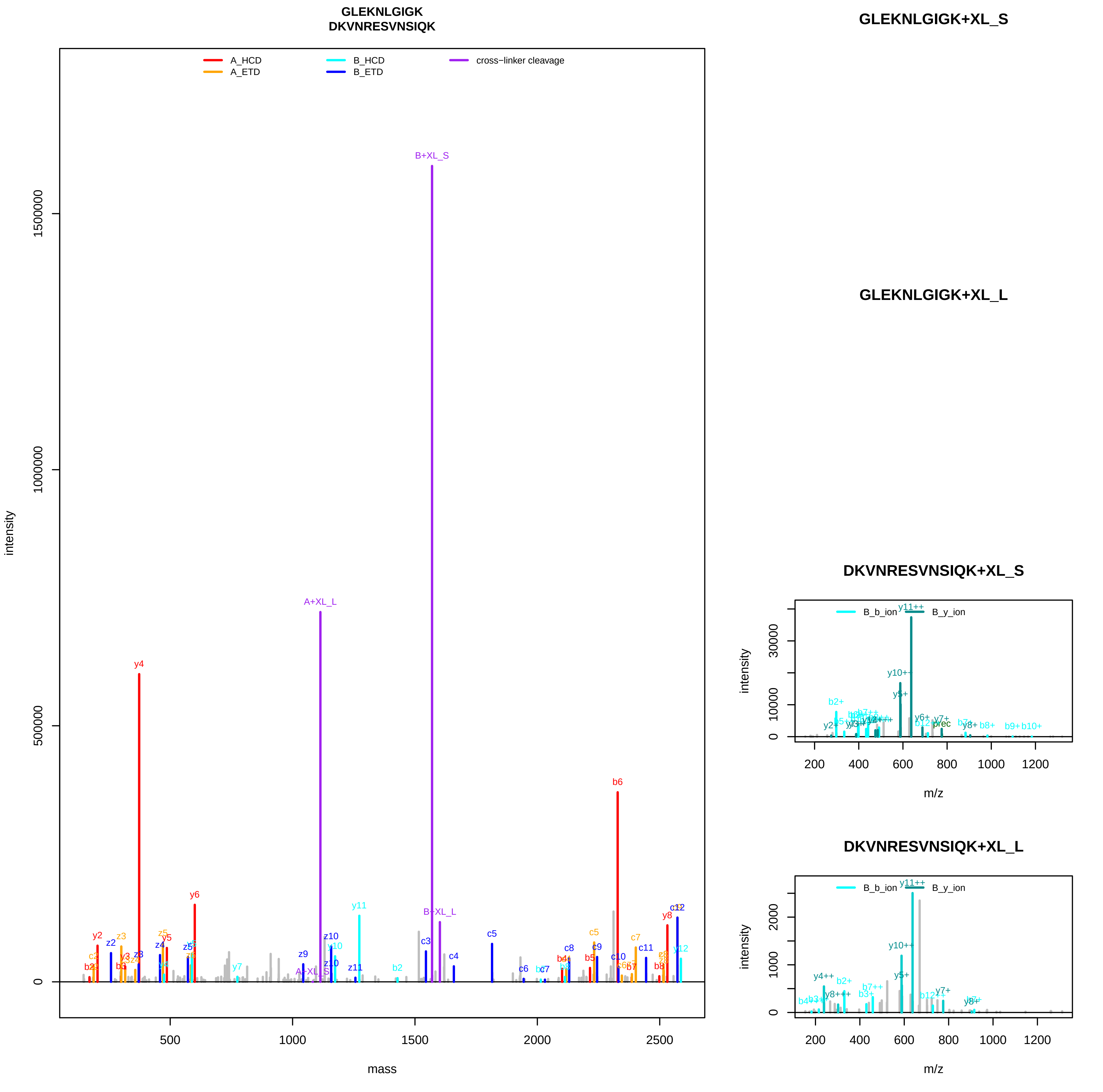

GLEKNLGIGK
DKVNRESVNSIQK
A_HCD
A_ETD
B_HCD
B_ETD
cross−linker cleavage
B+XL_S
A+XL_L
B+XL_L
A+XL_S
y4
y6
b6
y11
c5
c12
y8
intensity
mass
0
500000
1000000
1500000
500
1000
1500
2000
2500
GLEKNLGIGK+XL_S
GLEKNLGIGK+XL_L
DKVNRESVNSIQK+XL_S
B_b_ion
B_y_ion
y11++
y10++
y5+
b2+
intensity
m/z
0
10000
20000
30000
200
400
600
800
1000
1200
DKVNRESVNSIQK+XL_L
B_b_ion
B_y_ion
y11++
y10++
y5+
y4++
b2+
intensity
m/z
0
1000
2000
200
400
600
800
1000
1200
